# Supplementary material for: Development of a computer-aided design software for the quantitative evaluation of aesthetic damage
Source: PLoS One. 2019 Dec 18;14(12):e0226322. doi: 10.1371/journal.pone.0226322 (PMC6919621; doi:10.1371/journal.pone.0226322)
Supplement: S1 Code site — 1. (ZIP) [file pone.0226322.s001.zip › [EESC_jr][Projeto]Relatorio_07_21/reports/templates_zip/gentelella-master/production/invoice.html]

Gentellela Alela! | 


Gentellela Alela!

Welcome,

## John Doe

  


### General

- Home 
  - Dashboard
  - Dashboard2
  - Dashboard3
- Forms 
  - General Form
  - Advanced Components
  - Form Validation
  - Form Wizard
  - Form Upload
  - Form Buttons
- UI Elements 
  - General Elements
  - Media Gallery
  - Typography
  - Icons
  - Glyphicons
  - Widgets
  - Invoice
  - Inbox
  - Calendar
- Tables 
  - Tables
  - Table Dynamic
- Data Presentation 
  - Chart JS
  - Chart JS2
  - Moris JS
  - ECharts
  - Other Charts
- Layouts 
  - Fixed Sidebar
  - Fixed Footer

### Live On

- Additional Pages 
  - E-commerce
  - Projects
  - Project Detail
  - Contacts
  - Profile
- Extras 
  - 403 Error
  - 404 Error
  - 500 Error
  - Plain Page
  - Login Page
  - Pricing Tables
- Multilevel Menu 
  - Level One- Level One
      - Level Two
      - Level Two
      - Level Two
    - Level One
- Landing Page Coming Soon

- John Doe
  - Profile
  - 50%
    Settings
  - Help
  - Log Out
- 6
  - John Smith
    3 mins ago

    Film festivals used to be do-or-die moments for movie makers. They were where...
  - John Smith
    3 mins ago

    Film festivals used to be do-or-die moments for movie makers. They were where...
  - John Smith
    3 mins ago

    Film festivals used to be do-or-die moments for movie makers. They were where...
  - John Smith
    3 mins ago

    Film festivals used to be do-or-die moments for movie makers. They were where...
  - **See All Alerts**


### Invoice Some examples to get you started

Go!

## Invoice Design Sample user invoice design

- - Settings 1
  - Settings 2

# Invoice. Date: 16/08/2016

From
**Iron Admin, Inc.**
  
795 Freedom Ave, Suite 600
  
New York, CA 94107
  
Phone: 1 (804) 123-9876
  
Email: ironadmin.com

To
**John Doe**
  
795 Freedom Ave, Suite 600
  
New York, CA 94107
  
Phone: 1 (804) 123-9876
  
Email: jon@ironadmin.com

**Invoice #007612**
  
  
**Order ID:** 4F3S8J
  
**Payment Due:** 2/22/2014
  
**Account:** 968-34567


| Qty | Product | Serial # | Description | Subtotal |
| --- | --- | --- | --- | --- |
| 1 | Call of Duty | 455-981-221 | El snort testosterone trophy driving gloves handsome gerry Richardson helvetica tousled street art master testosterone trophy driving gloves handsome gerry Richardson | $64.50 |
| 1 | Need for Speed IV | 247-925-726 | Wes Anderson umami biodiesel | $50.00 |
| 1 | Monsters DVD | 735-845-642 | Terry Richardson helvetica tousled street art master, El snort testosterone trophy driving gloves handsome letterpress erry Richardson helvetica tousled | $10.70 |
| 1 | Grown Ups Blue Ray | 422-568-642 | Tousled lomo letterpress erry Richardson helvetica tousled street art master helvetica tousled street art master, El snort testosterone | $25.99 |

Payment Methods:


Etsy doostang zoodles disqus groupon greplin oooj voxy zoodles, weebly ning heekya handango imeem plugg dopplr jibjab, movity jajah plickers sifteo edmodo ifttt zimbra.

Amount Due 2/22/2014

|  |  |
| --- | --- |
| Subtotal: | $250.30 |
| Tax (9.3%) | $10.34 |
| Shipping: | $5.80 |
| Total: | $265.24 |


Print
 Submit Payment
 Generate PDF


Gentelella - Bootstrap Admin Template by Colorlib
